# Long-term sex differences in symptoms and immune profile in long COVID

**DOI:** 10.1186/s13293-026-00825-9

**Published:** 2026-01-27

**Authors:** José Feliz, Juliana Gonçalves, Carolina Cabedo, José Brito, Maria Gamas, Maria Inês Neves, Helena Soares

**Affiliations:** 1 Human Immunobiology and Pathogenesis Laboratory, iNOVA4Health, NOVA Medical School, Faculdade de Ciências Médicas, NOVA University of Lisbon, Lisbon, Portugal; 2 Unidade de Saúde Familiar (USF) Cuidar Saúde, Seixal, Portugal; 3 Egas Moniz Center for Interdisciplinary Research (CiiEM), Egas Moniz School of Health & Science, Caparica, Portugal

**Keywords:** Long COVID, Sex differential immunity, Long-term symptom persistence, Immune dysregulation, Cellular and molecular signatures

## Abstract

**Background:**

Long COVID (LC) is a post-infectious condition affecting millions worldwide, characterized by persistent multisystem symptoms. Females are disproportionately affected, reporting higher symptom burden, particularly neurocognitive and neurosensory complaints. While short-term immunopathology has been described, the long-term clinical course, immune dysregulation, and sex-specific underpinnings remain poorly understood.

**Methods:**

We analyzed 34 participants experiencing persisting symptoms from 9 months to 5 years post-SARS-CoV-2 infection, alongside 26 SARS-CoV-2–infected controls without symptoms. Clinical assessments, symptom inventories, comorbidity analysis, and work capacity evaluation were performed. Immune profiling included flow cytometry of CD4⁺ and CD8⁺ T cells, NK cells, and B cells, as well as quantification of plasma cytokines, soluble factors, and cytotoxic molecules, analyzed in a sex-disaggregated manner.

**Results:**

Females with LC exhibited higher symptom burden, particularly persistent fatigue, neurocognitive and neurosensory complaints, which increased with age and tended to increase with disease duration, whereas males showed no clear age- or duration-related patterns. Comorbidities, especially affecting endocrine, metabolic, and circulatory systems, were more frequent in females and aligned with symptom severity. Immune profiling revealed subtle but sex-specific differences: females had reduced CD8⁺ T cell cytotoxic profile, lower NKG2D and granzyme K expression, increased sCD40L and sFAS, and decreased perforin, whereas males displayed elevated TNF-α. NK cell function, B cells, and humoral immunity remained largely intact. Over half of participants reported functional impairments affecting work capacity.

**Conclusions:**

Even though our cohort is small it suggests that prolonged LC is characterized by sex-specific differences in symptom burden and immune profiles. Reduced cytotoxic CD8⁺ T cell profile in females may contribute to viral persistence and neurological symptoms, whereas elevated inflammatory markers in males suggest distinct immune pathways. These findings highlight the need for sex- and duration-specific management strategies, the identification of biomarkers, and the development of personalized therapies targeting specific LC endotypes.

**Supplementary Information:**

The online version contains supplementary material available at 10.1186/s13293-026-00825-9.

## Abstract (plain language summary)

Long COVID (LC) is a post-infectious condition affecting millions worldwide, marked by persistent, multisystem symptoms, with females disproportionately affected, particularly by neurocognitive and neurosensory complaints. Despite growing knowledge of short-term immune changes, the long-term clinical course, immune dysregulation, and sex-specific mechanisms remain poorly understood. In this study, we analysed 34 LC participants with persisting symptoms from 9 months to 5 years post-SARS-CoV-2 infection and compared them with 26 SARS-CoV-2–infected controls without symptoms. Participants underwent clinical assessment, symptom inventories, comorbidity analysis, and work capacity evaluation, while immune profiling included looking at specific immune cells and proteins in the blood. We looked into differences between males and females. We found that females with LC experienced more symptoms than males, especially fatigue, difficulty concentrating, and memory problems, and these symptoms tended to worsen with age and length of illness. Females also had more underlying health conditions, particularly related to metabolism, neurology, and circulation, which may contribute to their symptoms. Regarding the immune system, females showed changes in immune cells that help fight viruses, which could explain lingering symptoms and vulnerability to neurological issues, while males showed higher levels of general inflammation. More than half of participants reported difficulties with daily activities and work. These findings highlight that LC affects people differently based on sex, illness duration, and health history and underline the need for tailored approaches to treatment, better understanding of the disease, and strategies to support daily functioning and quality of life for those affected.

**Supplementary Information:**

The online version contains supplementary material available at 10.1186/s13293-026-00825-9.

## Highlights

- Prolonged Long COVID (LC) disproportionately affects females, who exhibit higher symptom burden, especially persistent fatigue and neurocognitive complaints, increasing with age and disease duration.
- Females with LC have more comorbidities, particularly in endocrine, metabolic, and circulatory systems, which may exacerbate symptoms through chronic inflammation and organ dysfunction.
- Immune profiling revealed sex-specific alterations: females display reduced CD8⁺ T cell cytotoxicity (lower granzyme K and NKG2D), increased sCD40L and sFAS, and decreased perforin, while males show elevated TNF-α.
- Over half of patients reported functional impairments affecting work capacity, underscoring the socioeconomic burden of LC.
- It emphasizes the need for sex- and duration-specific management strategies for LC.

**Supplementary Information:**

The online version contains supplementary material available at 10.1186/s13293-026-00825-9.

## Background

Five years have passed since the emergence of severe acute respiratory syndrome coronavirus 2 (SARS-CoV-2), and COVID-19 continues to pose a global health challenge through Long COVID (LC). The World Health Organization defines LC as a post-infectious condition with symptoms persisting for at least three months after COVID-19 onset, lasting a minimum of two months, and not explained by alternative diagnoses [1–4]. Globally, an estimated 65 million people are affected, with cases rising daily [5–7]. LC presents with a complex, multisystemic symptom profile, encompassing over 100 manifestations that range from mild to profoundly debilitating [8–12], including fatigue, cognitive impairment, respiratory and cardiovascular symptoms [13–21]. These symptoms often impair quality of life, limit daily functioning, and, in many cases, prevent patients from working [13, 22]. Although some recover, many individuals have experienced persistent symptoms since early 2020, with neurological sequelae such as brain fog and increased dementia risk reported to last for at least two years [23–31].

LC risk factors include female sex, older age, pre-existing comorbidities, ICU admission during acute COVID-19, lower socioeconomic status, and smoking [12, 13, 32, 33]. Biological sex strongly shapes LC presentation, mirroring other post-acute infection syndromes such as myalgic encephalomyelitis/chronic fatigue syndrome (ME/CFS) [34, 35]. Unlike acute COVID-19, where males face higher severity and mortality [36–39], LC disproportionately affects females, who report greater fatigue, neurocognitive deficits, headaches, and neurological sequelae, whereas males show more endocrine dysfunction [12, 40]. The role of biological sex in LC prevalence, symptomology, and underlying mechanisms has only recently begun to be explored [41, 42]. Investigating the cellular and molecular drivers of these sex differences may reveal critical insights into LC pathophysiology and inform precision therapeutic strategies.

While the short-term pathophysiology of Long COVID within the first-year post-diagnosis is increasingly understood [43–45], its long-term clinical course, immune alterations, and organ involvement remain largely unknown. Addressing this gap is critical, as LC can last two years or more [46, 47] and may, like ME/CFS, become a lifelong condition for some patients. To this end, we analyzed a cohort of 34 participants with LC, experiencing symptoms from 9 months up to 5 years post-infection, and characterized their clinical and immunological profiles in a sex-disaggregated manner.

## Methods

### Experimental model and subject details

A total of 34 Long COVID (LC) patients and 26 controls were recruited between June 2023 and March 2025. All LC patients underwent medical evaluations to exclude alternative medical aetiologies for their persistent symptoms. All participants were recruited at Medical Health Center USF Cuidar Saúde- Seixal. Demographic and clinical characteristics are detailed in Tables S1 and S2.

Blood samples were collected by venipuncture in EDTA tubes and were immediately processed. All participants signed the informed consent at the time of enrolment in the study and LC patients completed the clinical survey provided by the Portuguese General Directorate of Health (Direção Geral da Saúde). The survey included questions about COVID-19 data, namely acute disease severity (non-hospitalized or hospitalized), SARS-CoV-2 re-infection, vaccination status (number of doses, date and vaccine brand), employment status post infection and a list of 48 symptoms possible symptoms possible to be reported by LC patients.

All procedures were approved by NOVA Medical School ethics committee (178/2024/CEFCM) and by ethics committee for health from ARSLVT (2508/CES/2023), in accordance with the provisions of the Declaration of Helsinki and the Good Clinical Practice guidelines of the International Conference on Harmonization.

### Inclusion and exclusion criteria

Inclusion criteria for LC patients were age above 18 years old, previous positive PCR test for SARS-CoV-2 and persistent symptoms for at least 9 months after initial SARS-CoV-2 infection not attributed to any pre-existing comorbidity. Inclusion criteria for control group include age above 18 years and previous positive PCR test for SARS-CoV-2 with no active symptoms post infection.

Exclusion criteria for both groups include age below 18 years old and morbid obesity.

### Blood sample processing

Blood samples were first centrifuged at 1000 × g for 10 min to separate the plasma that was carefully removed and stored at -80 °C until further analysis. Peripheral blood mononuclear cells (PBMCs) were then isolated by density gradient centrifugation (Lymphosed, Biowest) [48–50] and cultured with RPMI supplemented with 10% FBS and 1% Antimycotic-Antibiotic, at 37 °C with 5% of CO2.

### Flow cytometry

PBMCs were stained with a fixable viability dye eFluor™ 506 (Invitrogen) and surface labelled with the following antibodies all from BioLegend: anti-CD3 (UCHT1), anti-CD4 (SK3), anti-CD8 (SK1), anti-CCR7 (G043H7), anti-CD45RA (HI100), anti-CD56 (HCD56), anti-NKG2D (1D11), anti-CD19 (SJ25C1), anti-CD27 (O323) and anti-CD38 (HIT2). Cells were washed, fixed with 1% PFA and permeabilized with Saponin. They were intracellular labelled with the following antibodies all from BioLegend: anti-Perforin (B-D48), anti-Granzyme B (GB11) and anti-Granzyme K (GM26E7). Cells were washed with FACS buffer and acquired in BD LSR Fortessa X-20 and analysed with FlowJo v10.7.3 software (Tree Star).

### ELISA

Antibody binding to SARS-CoV-2 trimeric spike protein or Nucleocapsid was assessed by a previously described in-house ELISA assay [48, 49, 51] based on the protocol by Stadlbauer et al. [52]. Briefly, 96-well plates (Nunc) were coated overnight at 4ºC with 0.5 $\upmu$g/ml of trimeric Spike or Nucleocapsid. After blocking with 3% BSA diluted in 0.05% PBS-T, 1:50 diluted plasma was added and incubated for 1 h at room temperature. Plates were washed and incubated for 30 min at room temperature with 1:25,000 dilution of HRP-conjugated anti-human IgA, IgG and IgM antibodies (Abcam, ab97225/ab97215/ab97205). Plates were washed and incubated with TMB substrate (BioLegend), stopped by adding phosphoric acid (Sigma) and read at 450 nm. Cut-off for plasma samples resulted from the mean of OD_450_ values from negative controls plus 3 times the standard deviation. Endpoint titers were established using a threefold dilution series starting at 1:50 and ending at 1:109,350 and defines as the last dilution before the signal dropped below OD_450_ of 0.15. This value was established using plasma from pre-pandemic samples collected from subjects not exposed to SARS-CoV-2. As previously described [51], in each assay we used 6 internal calibrators from 2 high-, 2 medium- and 2 low-antibody producers that had been diagnosed for COVID-19 through RT-PCR of nasopharyngeal and/or oropharyngeal swabs. As negative controls, we used pre-pandemic plasma samples collected prior to July 2019.

### Luminex

Plasma samples were thawed and tested in the LegendPlex Human panel (BioLegend) to quantify the levels of TNF-α, IL-10, perforin, sCD40L, sFAS, Granzyme A, Granzyme B, IL-6, IL-1b, IFN-γ, MCP-1, IL-18, and IL-23. The assay was performed according to manufactor’s instructions and was modified by using half of the amount of all reagents. All plasma samples were diluted 2X with assay buffer, and sample concentrations were calculated according to the dilution factor. Briefly, 12.5 µl of diluted plasma or standard, and 12.5 µl of mixed beads were added to each well and incubated for 2 h. The plate (V-bottom 96 well plate) was washed twice with 100 µl of wash buffer. Samples and standards were incubated with 12.5 µl of detection antibody for 1 h followed by an incubation of 30 min with 12.5 µl of Streptavidin-PE. The plate was washed once, and samples were resuspended in 75 µl of wash buffer. All incubation steps were performed at room temperature and protected from the light. Samples were acquired in a BD Accuri C6 plus (BD Biosciences) and analyzed with the Windows LegendPlex software (v8.0 BioLegend).

### Statistical analysis

Statistical analysis was performed using GraphPad Prism v9.00 and SPSS v26. First, we tested the normality of the data by using Shapiro Wilk (n < 6) or D’Agostino & Pearson (n > 6) normality tests, by checking skewness and kurtosis values and visual inspection of data. Then, if the samples followed a normal distribution, we chose the appropriate parametric test; otherwise, the non-parametric counterpart was chosen. In two groups comparisons: for unpaired data, Mann–Whitney test and the unpaired t test were used as indicated in figure legends. Spearman correlation test was used in correlation analysis as described in figure legends. Data represent mean ± SD for parametric tests, or median ± IQR for nonparametric tests. p value was considered significant at *p < 0.05, **p < 0.01, ***p < 0.001.

Fisher's Exact Test was used for categorical variables (Table S4). Logistic and Linear Regressions were used to adjust the results for sex, age and time since infection with Bonferroni adjustment (tables S4-S8).

To measure the magnitude of the difference, the effect size was calculated as described previously [53, 54]. For unpaired t tests: Cohen’s d is small if less than 0.3, medium if 0.3 or greater, or large if 0.8 or greater. For Mann–Whitney tests: correlation coefficient r is small if less than 0.3, medium if 0.3 or greater, or large if 0.5 or greater. Effect size values are reported for all figures in tables S9 and S10.

## Results

### Characterization of the study participants

**Fig. 1:**
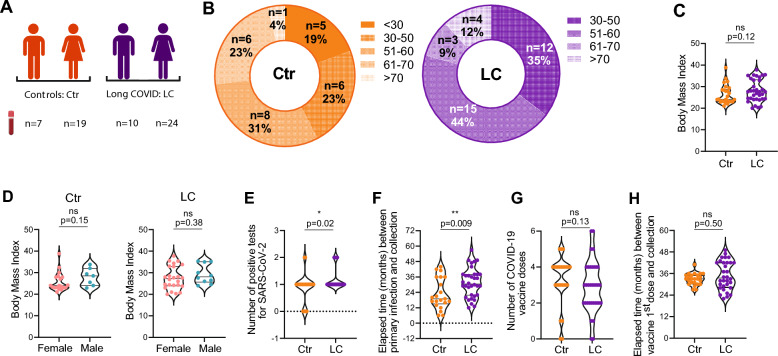
Characterization of study participants (**A**) Outline of participant recruitment, divided in 2 groups: controls (Ctr, orange) consisting of 7 males and 19 females, and Long COVID patients (LC, purple) consisting of 10 males and 24 females. (**B**) Distribution of Ctr (left) and LC (right) across the age intervals: < 30 years old (yo), 30-50 yo, 51-60 yo, 61-70 yo and > 70 yo. (**C**) Cumulative distribution of Body Mass Index (BMI) in Ctr (n = 26, orange) and LC patients (n = 34, purple). (**D**) Cumulative distribution of BMI disaggregated by sex in Ctr (left; females n = 19, males n = 7) and LC patients (right; females n = 24, males n = 10). (**E**) Number of SARS-CoV2 PCR-positive tests in Ctr (n = 26, orange) and LC patients (n = 34, purple). (**F**) Elapsed time (in months) between primary infection and sample collection in Ctr (n = 21, orange) and LC patients (n = 34, purple). (**G**) Number of COVID-19 vaccines doses received by Ctr (n = 26, orange) and LC patients (n = 34, purple). (**H**) Elapsed time (months) between the first COVID-19 vaccine dose and sample collection in Ctr (n = 24, orange) and LC patients (n = 32, purple). p values *p < 0.05, **p < 0.01; ns, not significant determined by non-parametric Mann-Whitney test (C, D, F-H) and by parametric unpaired t test (C, D). Effect sizes for all graphs are reported in Tables S9 and S10

Our study included 34 Long COVID (LC) patients recruited between June 2023 and March 2025, all of whom reported ongoing symptoms, including drowsiness, concentration difficulties, persistent fatigue, and insomnia, between 9 months and 5-years post SARS-CoV-2 infection. The cohort comprised 71% females and 29% males (Fig. 1A, Table S1), with sex designation assigned by the attending physician based on biological sex at birth. The median age was 53.5 years (interquartile range [IQR] 13), with 4 participants over 70 years of age (Fig. 1B, left). Seventy nine percent of patients were white and 21% were black. Only two participants required hospitalization for COVID-19, and one of these required supplemental oxygen (Table S1). The control group comprised 26 SARS-CoV-2–infected individuals without prolonged Long COVID, recruited concurrently with LC patients. The cohort was 73% female and 27% male, with a median age of 51.5 years (IQR 26) (Fig. 1A, Table S2).

The body mass index (BMI) ranged from 20 to 37.8 in LC patients (median 27.6, IQR 5.8) and 24.1 (IQR 5.5) in controls, with no significant differences between groups or by sex (Fig. 1C–D). Most participants had a single SARS-CoV-2 infection, though LC patients had more positive tests and a longer interval between primary infection and sample collection (Fig. 1E–F).

Regarding COVID-19 vaccination status, two LC patients and two controls had not received any COVID-19 vaccine at the time of sample collection (Tables S1–S2). Among controls, the majority (42%) had received four doses, whereas most LC patients had received two (29%) or three (26%) doses; no significant differences were observed between groups (Fig. 1G). The time elapsed between the first vaccine dose and sample collection was also similar across groups (Fig. 1H).

### Females with long COVID have more underlying health conditions

**Fig. 2:**
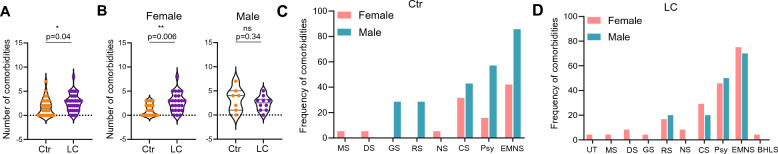
Sex-disaggregated comorbidities in persistent Long COVID patients. (**A**) Number of comorbidities in Ctr (n = 26) and in LC (n = 34). (**B**) Number of comorbidities in females (Ctr n = 19, LC n = 24) and in males (Ctr n = 7, LC n = 10). (**C**) Frequency of comorbidities in Ctr by health system. MS: Musculoskeletal System, DS: Digestive System, GS: Genital System, RS: Respiratory System, NS: Neurological System, CS: Circulatory System, P: Psychological, EMNS: Endocrine, Metabolic and Nutritional System. Females (n = 19) and Males (n = 7). (**D**) As in C, for LC. Females (n = 24) and males (n = 10). Data represents mean ± SD. p values *p < 0.05, **p < 0.01; ns, not significant determined by non-parametric Mann–Whitney test (A, B) and by parametric unpaired t test (B). Effect sizes for all graphs are reported in Tables S9 and S10

Comorbidities can impair the body’s ability to fight infections, and several studies have reported a higher prevalence of LC in individuals with chronic conditions [12, 13, 55–57]. Pre-existing comorbidities may also contribute to LC through an associated molecular profile, in which elevated inflammation drives oxidative stress, tissue damage, and organ dysfunction, thereby exacerbating and prolonging symptoms [13]. Nonetheless, we did not find an association between the number of comorbidities and LC status (Table S4). To assess whether comorbidity burden differed in our cohort and whether it was linked to sex, we analyzed the number of comorbidities in each group. We observed that LC patients had more comorbidities than controls (Fig. 2A). Sex-disaggregated analysis revealed that this difference was driven by females. Females with LC had more comorbidities than female controls (Fig. 2B, left), whereas no differences were observed between males (Fig. 2B, right).

To analyze participant comorbidities, we classified them into 10 health system categories. Overall, the most common comorbidities in controls were of circulatory system (CS; hypertension 9 participants, 35%), and of the endocrine, metabolic, and nutritional system (EMNS; obesity 6 participants, 23% and overweight 6 participants, 23%) (Table S3). In LC patients, comorbidities of the EMNS (obesity and lipid metabolism disorders, 14 patients each, 41%), CS (hypertension, 9 patients, 26%), and psychological conditions (Psy; depression, 8 patients, 24%) were the most prevalent (Table S3). A sex disaggregated analysis showed that the frequency of comorbidities is higher in control males than females in all the health system categories (Fig. 2C). Curiously, this difference was leveled in LC patients (Fig. 2D), with females exhibiting an increase in the frequency of EMNS and Psy comorbidities (Fig. 2D).

### Symptom burden is greater in females with long COVID.

**Fig. 3:**
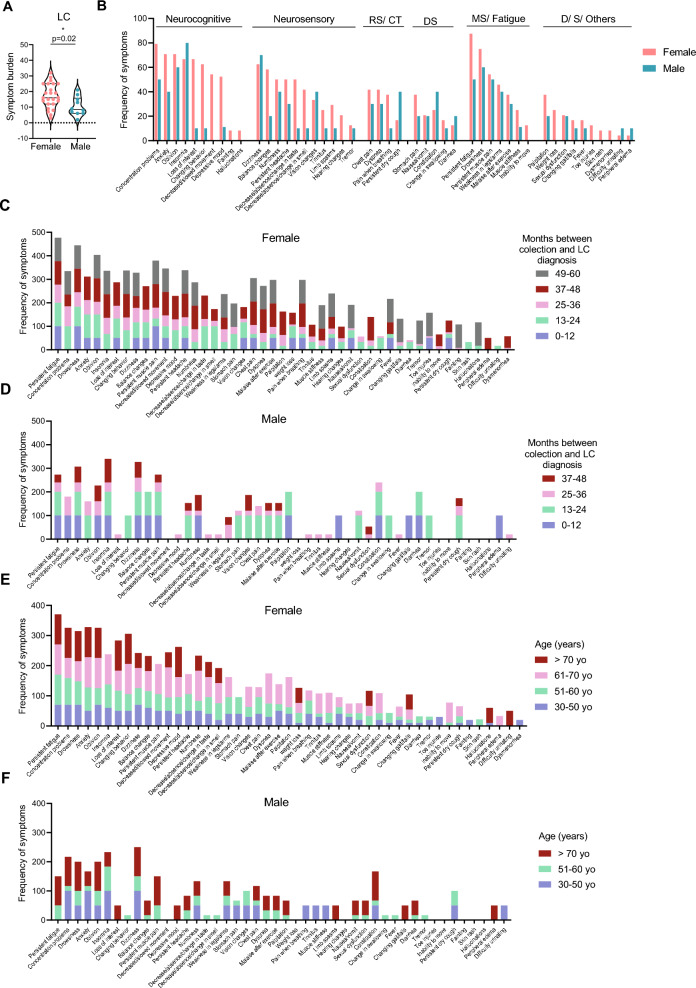
Sex-disaggregated symptomatology in persistent Long COVID patients (**A**) Symptom burden of LC patients disaggregated by sex. Females (n = 21) and males (n = 9). (**B**) Sex-disaggregated symptom frequency by category: Neurocognitive, Neurosensory, Respiratory System and Cardiothoracic (RS/CT), Digestive System (DS), Musculoskeletal System (MS) and Fatigue, Dermatological (D), Sexual (S) and Others. (**C**) Frequency of symptoms in LC females stratified by the elapsed time (months) between sample collection and diagnosis. (**D**) As in C, for LC males. (**E**) Age-wise distribution of symptoms in LC females. (**F**) As in E, for LC males. p value *p < 0.05 determined by parametric unpaired t test (A). Effect sizes for all graphs are reported in Table S9

LC symptoms significantly affect daily living, reducing quality of life and often causing disability [58–60]. We first assessed symptom burden by counting the number of self-reported symptoms per individual and found that females exhibited significantly higher symptom burden than males (Fig. 3A). In our cohort, the most frequently reported symptoms were persistent fatigue (76%, 26 patients), drowsiness, concentration problems, and insomnia (71%, 24 patients), followed by forgetfulness (68%, 23 patients), dizziness (65%, 22 patients), and anxiety (62%, 21 patients) (Figure S1A, Table S4). Less frequent symptoms, reported by only two patients (6%), included peripheral edema, difficulty urinating, fainting, skin rash, hallucinations and dysmenorrhea (Figure S1A, Table S3).

To examine sex differences, symptoms were grouped by health system categories and compared between females and males (Figs. 3B, S1B). Females reported higher frequencies of neurocognitive and neurosensory symptoms, including loss of interest, behavioral changes, depressive mood, slowed movements, and altered taste or smell. Of these, persistent fatigue positively associated with female sex (Table S5). Symptoms such as chest pain, nausea, and tremor were reported equally by both sexes (Fig. 3B).

We next evaluated the influence of time since LC diagnosis and patient age on symptom frequency. Females in our cohort had longer disease duration, with up to 60 months since diagnosis, compared with males, whose maximum duration was 48 months. Females diagnosed 37–60 months prior exhibited the highest symptom burden, whereas males with a 2-year diagnosis reported the most symptoms (Figs. 3C–D). However, we did not find an association between symptom burden and disease duration (Table S5). Symptom frequency in females was highest between 61 and 70 years with persistent fatigue positively associating with age (Table S5), while younger females reported fewer symptoms (Figs. 3E–F). No clear age-related pattern was observed in males (Figs. 3E–F). It should be noted that no male participants were included in the 61–70-year age group, which limits direct sex-based comparisons within this age stratum. Among the 30 LC patients who responded, more than half (63%) reported negative impacts on work (Figure S1C), with 53% of these indicating a need to stop working entirely.

Overall, our results suggest that females with LC experience a higher symptom burden than males, particularly for persistent fatigue, neurocognitive and neurosensory symptoms. In females, symptom frequency increases with older age, whereas no clear age-related pattern is observed in men. LC also substantially affects daily functioning, including work capacity.

## CD8^+^ T cells display sex differential cytotoxic phenotype in LC patients 

Although T cells play a critical role in SARS-CoV-2 clearance and recovery from COVID-19 [61–65], their involvement in LC remains unclear [66, 67]. Some studies have reported T cell alterations, including exhausted T cells [42], reduced CD4⁺ and CD8⁺ memory cells [42, 68], and elevated PD-1 expression on central memory cells [69], whereas others found no differences in total CD4⁺ or CD8⁺ T cells or their memory compartments [66, 70, 71]. To investigate cellular immune changes in LC, we phenotyped CD4⁺ and CD8⁺ T cells by flow cytometry and compared their profiles between controls and LC patients. CD4⁺ T cell profiles, including memory subsets and activation markers, were also examined (Figures S2A, S3A–E). Consistent with previous reports [66, 70, 71], total CD4⁺ T cell levels and activation status were similar in controls and LC patients (Figures S3A–B). Memory subsets, defined by CCR7 and CD45RA expression, were also comparable between groups, including T effector memory cells (CCR7⁻ CD45RA⁻, Figure S3C), T central memory cells (CCR7⁺ CD45RA⁻, Figure S3D), and T memory cells re-expressing RA (CCR7⁻ CD45RA⁺, Figure S3E).

**Fig. 4:**
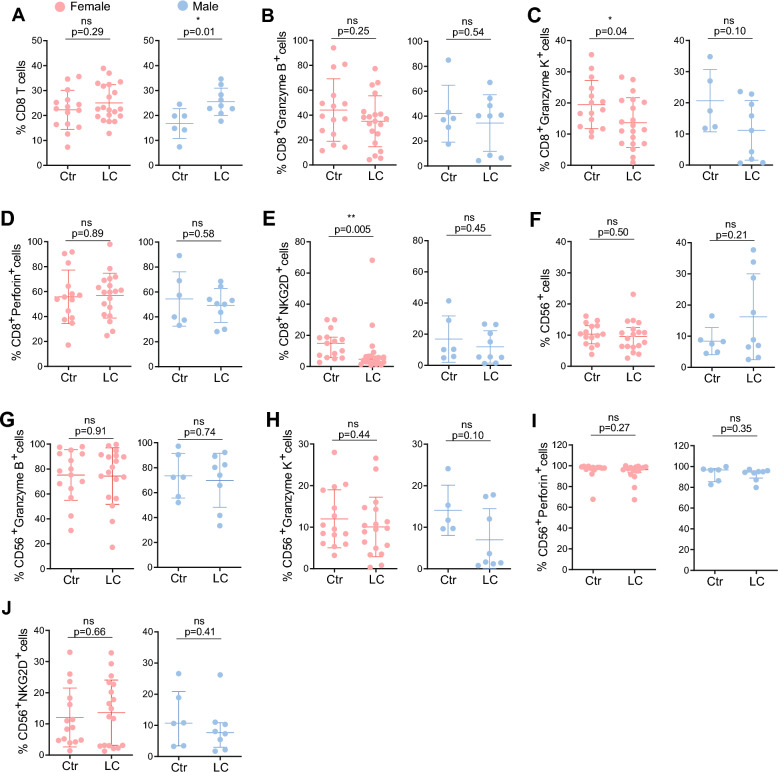
Sex-disaggregated cytotoxic response in persistent Long COVID patients. (**A**) Frequency of CD8^+^ T cells in females (left; Ctr n = 15, LC n = 20) and males (right; Ctr n = 6, LC n = 9). (**B**) Frequency of CD8^+^Granzyme B^+^ cells in females (left; Ctr n = 15, LC n = 20) and males (right; Ctr n = 6, LC n = 9). (**C**) Frequency of CD8^+^Granzyme K^+^ cells in females (left; Ctr n = 15, LC n = 20) and males (right; Ctr n = 5, LC n = 9). (**D**) Frequency of CD8^+^Perforin^+^ cells in females (left; Ctr n = 14, LC n = 19) and males (right; Ctr n = 6, LC n = 9). (**E**) Frequency of CD8^+^NKG2D^+^ cells in females (left; Ctr n = 15, LC n = 20) and males (right; Ctr n = 6, LC n = 9) (**F**) Frequency of CD56^+^ cells in females (left; Ctr n = 15, LC n = 18) and males (right; Ctr n = 6, LC n = 9). (**G**) Frequency of CD56^+^Granzyme B^+^ cells in females (left; Ctr n = 15, LC n = 18) and males (right; Ctr n = 6, LC n = 8). (**H**) Frequency of CD56^+^Granzyme K^+^ cells in females (left; Ctr n = 15, LC n = 18) and males (right; Ctr n = 5, LC n = 8). (**I**) Frequency of CD56^+^Perforin^+^ cells in females (left; Ctr n = 14, LC n = 17) and males (right; Ctr n = 6, LC n = 8). (**J**) Frequency of CD56^+^NKG2D^+^ cells in females (left; Ctr n = 15, LC n = 18) and males (right; Ctr n = 6, LC n = 8). Data represents mean ± SD for parametric tests, or median ± IQR for nonparametric tests. p values *p < 0.05, **p < 0.01; ns, not significant determined by parametric unpaired t test (A-H, J) and by non-parametric Mann-Whitney test (E, F, I, J). Effect sizes for all graphs are reported in Tables S9 and S10

To characterize the cytotoxic profile of CD8⁺ T cells, we assessed expression of granzyme B and K, perforin, and Natural Killer group 2 member D (NKG2D) by flow cytometry (Figure S2B). Given that sex can influence LC presentation (Fig. 3) and progression [41, 42, 72], we disaggregated our analysis by sex (Fig. 4A–E). The frequency of CD8⁺ T cells was slightly increased in the LC group (Figure S4A), with sex-disaggregated analysis revealing that this increase was driven by males (Fig. 4A).

Examination of the cytotoxic profile showed no sex differences in granzyme B (Fig. 4B) or perforin (Fig. 4D), and no significant differences were observed between controls and LC patients (Figures S4E, S4G), consistent with previous reports [66, 70]. Similarly, the frequencies of CD8⁺ effector memory, central memory, and TEMRA subsets did not differ significantly between groups (Figures S4B–D). Interestingly, the production of granzyme K by CD8⁺ T cells was significantly decreased in LC patients, a change that appeared to be driven primarily by females (Figures SFC, 4C), even though it did not reach statistical significance (Table S6). Similarly, NKG2D expression was reduced in LC patients, with this reduction also seemingly attributable to females (Figures S4H, 4E), even though statistical significance was not reached (Table S6).

Next, we examined Natural Killer (NK) cells, which play a critical role in innate antiviral defense and may become impaired during acute COVID-19, reducing their ability to eliminate infected cells [73]. However, their role in post-acute infection, and particularly in LC, remains unclear [68, 73]. First, we assessed overall NK cell (Fig S2C) frequency consistent with previous reports [68, 73], we found no differences between controls and LC patients, [68, 73] nor between females and males (Figures S4I, 4F). As for the expression of granzyme B, granzyme K, perforin, and NKG2D (Figs. 4G–J, S4J–M) by NK cells, no significant differences were observed between controls and LC patients for any of the cytotoxic markers (Table S7).

Lastly, we assessed humoral immunity, as antibodies generated after natural SARS-CoV-2 infection provide protection against reinfection [74, 75], even though the role of B cells in LC is still a subject of active research [76, 77]. No significant differences were observed in total B cells, memory B cells, or plasmablasts between groups (Figures S2D, S3F–H). Consistent with prior studies [22, 70, 78], we found no differences in IgA, IgG, or IgM antibodies against the Spike or Nucleocapsid proteins between controls and LC patients (Figures S3I–N).

Our results indicate that LC is associated with subtle, sex-specific alterations in CD8⁺ T cell cytotoxic profile, particularly reduced granzyme K and NKG2D in women, while B cells, CD4⁺ T cells, NK cells, and humoral immunity remain largely unaffected. This highlights a potential role for CD8⁺ T cell dysfunction in female patients as a contributor to persistent LC pathophysiology.

### Long COVID patients exhibit sex-specific differences in systemic inflammatory profiles.

**Fig. 5:**
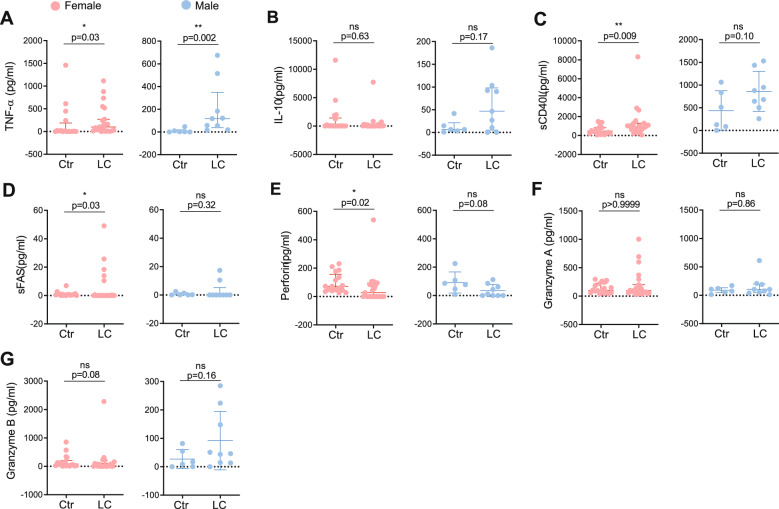
Sex-disaggregated inflammatory plasma profile in Long COVID patients (**A**) Plasma concentration of TNF-α (pg/ml) in females (left; Ctr n = 16, LC n = 23) and males (right; Ctr n = 6, LC n = 9). (**B**) Plasma concentration of IL-10 (pg/ml) in females (left; Ctr n = 18, LC n = 23) and males (right; Ctr n = 6, LC n = 9). (**C**) Plasma concentration of sCD40L (pg/ml) in females (left; Ctr n = 18, LC n = 22) and males (right; Ctr n = 6, LC n = 9). (**D**) Plasma concentration of sFAS (pg/ml) in females (left; Ctr n = 18, LC n = 23) and males (right; Ctr n = 6,LC n = 9). (**E**) Plasma concentration of Perforin (pg/ml) in females (left; Ctr n = 17, LC n = 23) and males (right; Ctr n = 6, LC n = 9). (**F**) Plasma concentration of Granzyme A (pg/ml) in females (left; Ctr n = 18, LC n = 23) and males (right; Ctr n = 6, LC n = 9). (**G**) Plasma concentration of Granzyme B (pg/ml) in females (left; Ctr n = 18, LC n = 23) and males (right; Ctr n = 6, LC n = 9). Data represents mean ± SD for parametric tests, or median ± IQR for nonparametric tests. p values *p < 0.05, **p < 0.01; ns, not significant determined by non-parametric Mann-Whitney test (A-G) and by parametric unpaired t test (C, E, G). Effect sizes for all graphs are reported in Tables S9 and S10

Recent studies have sought to link specific markers of immune dysfunction and inflammation with LC [79–82]. Cytokines such as interleukin (IL)-1β, IL-6, tumor necrosis factor (TNF-α), and interferon-gamma-inducible protein (IP-10) have been repeatedly associated with the condition [83–87]. To investigate systemic inflammation in LC, we quantified plasma cytokines in a sex-disaggregated manner. Consistent with prior reports [84, 86, 87], TNF-α levels were elevated in LC patients (Figure S5A). Although TNF-α was also increased in females with LC compared to female controls, the elevation was more pronounced in males (Fig. 5A), but without reaching statistical significance (Table S8). In contrast, anti-inflammatory IL-10 levels did not differ significantly between groups (Figs. 5B, S5B). sCD40L, a marker linked to inflammation [88] and vascular risk [89], was elevated in LC patient serum, with the increase predominantly observed in females (Figs. 5C, S5C). However, it did not reach statistical significance (Table S8). Soluble Fas (sFAS) has been detected in the serum of COVID-19 patients, with levels correlating with disease severity [90]. In our cohort, sFAS levels were modestly increased in LC patients, driven primarily by females, who showed higher circulating sFAS than female controls (Figs. 5D, S5D), without reaching statistical significance (Table S8). As for the plasma levels of IL-6, IL-1b, IFN-γ, MCP-1, IL-18, and IL-23 they were similar between controls and LC groups (Figure S5 H-M).

To examine the cytotoxic profile in LC patients, we measured plasma concentrations of perforin and granzymes A and B. Serum perforin was reduced in LC patients, driven by females, whereas granzymes A and B levels remained unchanged across groups and sexes (Figs. 5E–G, S5E–G, Table S8).

Next, we investigated whether the time elapsed between LC diagnosis and sample collection influenced plasma levels of cytokines, soluble factors, or granzymes by performing correlation analyses (Figures S5H–M). Interestingly, TNF-α, IL-10, sCD40L, sFAS, and granzyme A showed no correlation with disease duration (Figures S5H–K, S5M). Perforin was the only factor displaying a positive correlation, with levels increasing in patients with longer disease duration, although the correlation was modest (Figure S5L).

Collectively, our results suggest that LC is associated with a sex-differential inflammatory profile. TNF-α levels are elevated, particularly in males, while females show increased sCD40L and sFAS, alongside reduced perforin, further supporting a potential impairment of cytotoxic function (Fig. 4C). Other immune mediators, including IL-10 and granzymes A and B, remain unchanged.

## Discussion

LC is an emerging syndrome, affecting around 65 million people worldwide [6, 7]. The world has been confronted with new SARS-CoV-2 variants, leading to repeated infections [91] and conducting to a high prevalence of LC [92]. Due to LC heterogeneity, the plethora of relapsing and remitting symptoms [93] and multisystemic organ involvement [10, 11], the exact mechanisms underpinning LC pathophysiology and long-term prognosis remain unclear. Here, we sought to contribute to filling this knowledge gap, by studying a cohort of 34 patients with LC. In our cohort, females experienced a higher symptom burden, particularly persistent fatigue and neurocognitive and neurosensory manifestations, alongside a propensity for greater comorbidity load, which increased with age. Males, in contrast, tended to display stronger systemic inflammatory signals, notably elevated TNF-α, but fewer symptoms overall. Immune profiling revealed sex-dependent alterations in the cytotoxic profile of CD8⁺ T cells, with females showing a tendency for reduced granzyme K and NKG2D expression, increased sCD40L and sFAS, and decreased perforin, while other lymphocyte populations and humoral responses remained largely unaffected. These findings highlight potentially distinct pathogenic mechanisms in males and females and underscore the need for sex-tailored approaches in the management and study of LC.

The exact etiology of LC remains unclear, but several risk factors, including female sex, age, comorbidities, and lower socioeconomic status, have been identified [12, 13, 32, 33]. Consistent with these associations, LC patients in our cohort had a propensity for higher number of comorbidities than controls, with females showing the greatest burden. Co-morbidities affecting females were enriched in the endocrine, metabolic, psychological, and circulatory systems, which may exacerbate disease through sustained inflammation, oxidative stress, and organ dysfunction. These comorbidities likely contribute both to the onset and persistence of LC, in line with previous studies [12, 13, 55–57]. Symptom analysis revealed that females reported higher frequencies of chronic fatigue among other neurocognitive and neurosensory complaints, such as drowsiness, concentration deficits, and altered taste or smell, consistent with prior observations [42, 94]. The pattern of neurocognitive and neurosensory symptoms resembles myalgic encephalomyelitis/chronic fatigue syndrome (ME/CFS), a condition characterized by severe fatigue, post-exertional malaise, and cognitive impairment, with a female-to-male prevalence ratio of approximately 4:1 [95]. Viral infections, such as by Epstein-Barr virus, have been implicated in ME/CFS, and emerging evidence suggests that SARS-CoV-2 may similarly trigger ME/CFS in a subset of patients [96]. Moreover, symptom burden in females increased both age and tendentially with longer disease duration, whereas males showed no clear age- or duration-dependent patterns, emphasizing the need for sex- and duration-specific management strategies. Importantly, more than half of responding patients reported negative impacts on work, with many needing to stop working entirely, highlighting the substantial functional and socioeconomic burden of LC.

Our immune profiling revealed subtle but sex-specific alterations in CD8⁺ T cell cytotoxic profile in LC patients. While total CD8⁺ T cell levels tended to be slightly increased in males, females exhibited a propensity for reduced granzyme K and NKG2D expression, which might indicate impaired cytotoxic function. The overall increase in CD8⁺ T cells in patients may reflect ongoing immune responses against a persistent SARS-CoV-2 reservoir in multiple organs, as these cells are essential for viral clearance [97]. Alternatively, it is also possible that other chronic viral infections may contribute to sustained CD8⁺ T-cell activation and expansion of effector populations, particularly in older adults. Prior work has shown that LC symptoms, such as fatigue and neurocognitive dysfunction, at a median of four months post-diagnosis were independently associated with serological evidence of recent EBV reactivation or elevated EBNA IgG levels, but not with ongoing EBV viremia [98]. Interestingly, prior CMV infection appeared protective against neurocognitive LC [98], suggesting that antigenic burden and immune imprinting from latent infections may modulate clinical outcomes. Moreover, associations between LC and VZV reactivation have also been reported [99]. These observations underscore the complexity of immune activation in LC and highlight the need for future studies to incorporate comprehensive assessments of latent viral co-infections (e.g., CMV, EBV, VZV) and their potential synergistic or antagonistic effects on T-cell phenotypes and symptom persistence. The fact that the increase in CD8 + T cell frequency tended to be driven primarily by males may help explain why male sex is not a major risk factor for developing LC. NKG2D, a key activating receptor on CD8⁺ T and NK cells, acts as a co-stimulatory molecule in CD8⁺ T cells, enhancing cytotoxicity and contributing to long-term memory formation [100, 101]. Blockade of NKG2D has been shown to impair memory CD8⁺ T cell development [102]. In our cohort, despite the higher total CD8⁺ T cell counts, LC patients, particularly females, tendentially showed reduced NKG2D granzyme K expression, and perforin levels in the plasma. These alterations may indicate differences in CD8⁺ T cell functional capacity that could contribute to less efficient clearance of infected cells and potentially influence the development of long-term memory T cells. Although LC patients exhibited a trend toward reduced central memory CD8⁺ T cells compared to controls, this difference did not reach statistical significance (p = 0.09). These findings suggest that any impairment in CD8⁺ memory formation may be subtle and not readily detectable in cohorts of this size. Future studies incorporating larger sample sizes and additional functional markers, such as PD-1, TOX, TIM-3, and TIGIT, will be essential to clarify whether CD8⁺ T-cell exhaustion or memory dysregulation contributes to the immunopathology of LC. While plasma perforin does not directly reflect cellular cytotoxicity, several studies have nonetheless reported associations between plasma perforin levels and clinical outcomes influenced by cytotoxic immune activity, including responses to cancer therapies [103] and chronic hepatitis B treatment [104]. Plasma perforin has also been proposed as a serologic marker of clinical stage in virus-induced severe fever with thrombocytopenia syndrome [105]. Consistent with previous studies, we observed no differences in the frequency of CD4^+^ T cells or in their memory subsets [70, 71]. Yet, the specific role of these cells in LC etiology is still to be clarified [66, 67], as other authors have found alterations in central memory and exhausted profile [42, 68, 69]. NK cells, critical components of the innate antiviral response, displayed no significant differences in overall frequency or cytotoxic marker expression (granzyme B, granzyme K, perforin, NKG2D) between LC patients and controls, or between sexes. This suggests that, in contrast to CD8⁺ T cells, NK cell cytotoxic profile is largely preserved in LC. Similarly, humoral immunity appeared intact. No differences were observed in total B cells, memory B cells, plasmablasts, or in antibody responses (IgA, IgG, IgM) against SARS-CoV-2 Spike and Nucleocapsid proteins between LC patients and controls. Indicating that LC is not associated with major deficits in the B cell compartment or antibody-mediated immunity. These findings may suggest a potential role for CD8⁺ T cell dysfunction in the pathophysiology of LC, particularly in female patients.

Systemic inflammatory markers also tended to exhibit sex-specific patterns. TNF-α levels were elevated predominantly in males, whereas females showed increased sCD40L and sFAS alongside with reduced perforin. Unlike other chronic infection-associated conditions such as ME/CFS, where TNF-α declines after 2–3 years [106], TNF-α levels in LC remained stable over disease duration. Soluble CD40L levels displayed a propensity to be elevated in LC patients, reflecting patterns seen in autoimmune and inflammatory diseases, where sCD40L amplifies systemic inflammation [88, 107–109] and can disrupt the blood–brain barrier [109]. Similarly, increased sFAS, which can interfere with apoptosis and the clearance of damaged or infected cells [90], suggests ongoing immune activation in women and may further exacerbate chronic symptoms. Together, these observations indicate that LC might be associated with systemic inflammation and cytotoxic dysfunction, with sex-specific patterns that may underlie the female bias in symptom severity and neurological manifestations.

Understanding the mechanisms driving LC will not only advance LC research but also provide valuable insights into other infection-associated chronic conditions, including ME/CFS and chronic Lyme disease. Our findings add to the growing evidence that sex-differential immunity plays a key role in shaping disease trajectories, highlighting the urgent need for tailored therapeutic strategies.

This study has several limitations. The major one is that our LC cohort was relatively small and lacked ethnic and racial diversity, which places some constraints in the robustness of our observations and may contribute to explain the modest results of our linear and logistic regression analysis. In addition, baseline clinical parameters prior to acute infection were not available, limiting our ability to assess pre-existing differences. The absence of male participants in the 61–70-year age group represents a limitation when interpreting age-stratified symptom comparisons between sexes. This imbalance may introduce bias in assessing sex-related differences within older cohorts and restricts the generalizability of our findings. Future studies should aim to recruit more balanced cohorts across age and sex strata to enable robust stratified analyses and minimize potential confounding effects. Another limitation is the potential limitation by patients to attend healthcare consultations, due to severe illness, reduced mobility, or social exclusion, were not captured, which may have introduced selection bias. Despite these limitations, our study provides a detailed, sex-disaggregated characterization of LC patients, combining clinical, immunological, and inflammatory profiling. Lastly, we must acknowledge the potential confounding role of comorbidities in shaping immune signatures and the subsequent limitations of fully disentangling LC-specific effects from comorbidity-driven influences. Nonetheless, the inclusion of a well-matched SARS-CoV-2–infected control group strengthens our comparative analyses. Moreover, by examining long-term LC patients, some with symptoms persisting over four years, we offer unique insights into chronic disease mechanisms, immune dysfunction, and sex-specific differences that can inform future therapeutic strategies.

### Perspectives and significance

Our findings highlight the complex, sex-specific nature of LC. Together, these results emphasize the need for sex- and duration-specific management strategies, the identification of robust biomarkers, and the development of personalized therapies targeting distinct LC endotypes. Our results provide mechanistic insights into symptom persistence and immune dysregulation, highlighting females’ heightened vulnerability. Looking forward, these insights could inform the design of clinical trials by encouraging sex-stratified analyses and testing therapies that restore cytotoxic T cell function, including immunomodulatory or antiviral strategies.

## Conclusions

Females tended to exhibit heightened symptom burden, particularly neurocognitive and neurosensory complaints, which increased with age and disease duration, whereas men show no clear age- or duration-dependent patterns. Comorbidities, especially affecting endocrine, metabolic, and circulatory systems, likely exacerbate these symptoms through sustained inflammation and organ dysfunction. At the immune level, we observed subtle sex differences: women with LC had a propensity to display reduced CD8⁺ T cell cytotoxic function, lower NKG2D and granzyme K expression, increased sCD40L and sFAS, and decreased perforin, whereas men show elevated TNF-α levels. These molecular signatures may contribute to symptom persistence, immune dysregulation, and neurological manifestations in women. Importantly, over half of patients reported functional impairments affecting work capacity, underscoring the socioeconomic burden of LC. By integrating clinical, immunological, and inflammatory profiling in a sex-disaggregated manner, this work emphasizes the importance of personalized, sex- age- and duration-specific management strategies.

## Supplementary Information

Supplementary Materials 1

Supplementary Materials 2: **Fig. S1 **Frequency of symptoms in Long COVID patients. (**A**) Frequency of symptoms of LC patients. (**B**) Frequency of symptoms disaggregated by sex (females, pink; males, blue). (**C**) Work-related impacts of LC

Supplementary Materials 3: **Fig. S2 **Flow cytometric gating strategy. (**A**) Gating strategy for activated and memory CD4+ T cell subsets. (**B**) Gating strategy for Granzyme B^+^, Granzyme K^+^, Perforin^+^ and NKG2D^+^ CD8^+^ T cells and memory CD8^+^ T cell subsets. (**C**) Gating strategy for Granzyme B^+^, Granzyme K^+^, Perforin^+^ and NKG2D^+^ CD56^+^ cells. (**D**) Gating strategy for CD19^+^CD3^−^CD27^+^ and CD19^+^CD3^−^CD27^+^CD38^+^ B cells

Supplementary Materials 4: **Fig. S3 **Humoral and cellular responses in Long COVID. (**A**) Frequency of CD4^+^ T cells in Ctr (n = 20) and LCs (n = 15). (**B**) Frequency of CD4^+^ CD38^+^ T cells in Ctr (n = 19) and LCs (n = 13). (**C**) Frequency of CD4^+^ T effector memory (TEM) cells in Ctr (n = 15) and LCs (n = 8). (**D**) Frequency of CD4^+^ T central memory (TCM) cells in Ctr (n = 15) and LCs (n = 8). (**E**) Frequency of CD4^+^ TMRA cells in Ctr (n = 15) and LCs (n = 8). (**F**) Frequency of circulating B cells in Ctr (n = 20) and LCs (n = 15). (**G**) Frequency of memory B cells in Ctr (n = 20) and LCs (n = 11). (**H**) Frequency of plasmablasts B cells in Ctr (n = 19) and LCs (n = 13) (**I**) Anti-spike IgA endpoint titer (Ctr n = 26; n = 34). (**J**) Anti-spike IgG endpoint titer (Ctr n = 26; LC n = 34). (**K**) Anti-spike IgM endpoint titer (Ctr n = 26; LC n = 34). (**L**) Anti-Nucleocapsid IgA endpoint titer (Ctr n = 26; LC n = 34). (**M**) Anti-spike IgG endpoint titer (Ctr n = 26; LC n = 34). (**N**) Anti-spike IgM endpoint titer (Ctr n = 26; LC n = 34). Data represents mean ± SD for parametric tests, or median ± IQR for nonparametric tests. nd: not detectable; p values ns, not significant determined by parametric unpaired t test (A, C, D) and by non-parametric Mann-Whitney test (B, E-N). Effect sizes for all graphs are reported in Tables S9 and S10.

Supplementary Materials 5: **Fig. S4 **CD8⁺ T cell and NK cell characterization in controls and Long COVID patients. (**A**) Frequency of CD8^+^ T cells in Ctr (n = 21) and LC (n = 29). (**B**) Frequency of CD8^+^ T effector memory (TEM) cells in Ctr (n = 21) and LCs (n = 26). (**C**) Frequency of CD8^+^ T central memory (TCM) cells in Ctr (n = 21) and LCs (n = 26). (**D**) Frequency of CD8^+^ TMRA cells in Ctr (n = 21) and LCs (n = 26). (**E**) Frequency of CD8^+^Granzyme B^+^ cells in Ctr (n = 21) and LC (n = 29). (**F**) Frequency of CD8^+^Granzyme K^+^ cells in Ctr (n = 20) and LC (n = 29). (**G**) Frequency of CD8Perforin^+^ cells in Ctr (n = 20) and LC (n = 28). (**H**) Frequency of CD8^+^ NKG2D^+^ cells in Ctr (n = 21) and LC (n = 29). (**I**) Frequency of CD56^+^ cells in Ctr (n = 21) and LC (n = 27). (**J**) Frequency of CD56^+^Granzyme B^+^ cells in Ctr (n = 21) and LC (n = 26). (**K**) Frequency of CD56^+^Granzyme K^+^ cells in Ctr (n = 20) and LC (n = 26). (**L**) Frequency of CD56^+^Perforin^+^ cells in Ctr (n = 20) and LC (n = 25). (**M**) Frequency of CD56^+^ NKG2D^+^ cells in Ctr (n = 21) and LC (n = 26). Data represents mean ± SD for parametric tests, or median ± IQR for nonparametric tests. p values *p < 0.05, **p < 0.01; ns, not significant determined by parametric unpaired t test (A, B, D-G, J, K, M) and by non-parametric Mann–Whitney test (C, H, I, L). Effect sizes for all graphs are reported in Tables S9 and S10

Supplementary Materials 6: **Fig. S5** Inflammatory plasma profile in controls and persistent Long COVID patients (**A**) Plasma concentration of TNF-α (pg/ml) in Ctr (n = 22) and LC (n = 32). (**B**) Plasma concentration of IL-10 (pg/ml) in Ctr (n = 24) and LC (n = 32). (**C**) Plasma concentration of sCD40L (pg/ml) in Ctr (n = 24) and LC (n = 30). (**D**) Plasma concentration of sFAS (pg/ml) in Ctr (n = 24) and LC (n = 32). (**E**) Plasma concentration of Perforin (pg/ml) in Ctr (n = 24) and LC (n = 32). (**F**) Plasma concentration of Granzyme A (pg/ml) in Ctr (n = 24) and LC (n = 32). (**G**) Plasma concentration of Granzyme B (pg/ml) in Ctr (n = 24) and LC (n = 32). (**H**) Plasma concentration of IL-1β (pg/ml) in Ctr (n = 20) and LC (n = 32). (**I**) Plasma concentration of IFN-$\upgamma$ (pg/ml) in Ctr (n = 24) and LC (n = 32). (**J**) Plasma concentration of MCP-1 (pg/ml) in Ctr (n = 18) and LC (n = 21). (**K**) Plasma concentration of IL-6 (pg/ml) in Ctr (n = 23) and LC (n = 32). (**L**) Plasma concentration of IL-18 (pg/ml) in Ctr (n = 24) and LC (n = 32). (**M**) Plasma concentration of IL-23 (pg/ml) in Ctr (n = 24) and LC (n = 32). (**N**) Correlation between plasma TNF-α concentration (pg/mL) and elapsed time (months) between LC diagnosis and sample collection (n = 32). (**O**) As in H for IL-10 (n = 32). (**P**) As in H for sCD40L (n = 30). (**Q**) As in H for sFAS (n = 32). (**R**) As in H for Perforin (n = 32). (**S**) As in H for Granzyme A (n = 32). Data represents median ± IQR for nonparametric tests. p values *p < 0.05, **p < 0.01, ***p < 0.0001; ns, not significant determined by non-parametric Mann-Whitney test (A-M). Spearman correlation (N-S). Effect sizes for all graphs are reported in Table S9.

## Data Availability

The datasets used and/or analyzed during the current study are available from the corresponding author on reasonable request.
