# Supplementary material for: Long-term sex differences in symptoms and immune profile in long COVID
Source: Biol Sex Differ. 2026 Jan 27;17:32. doi: 10.1186/s13293-026-00825-9 (PMC12918094; doi:10.1186/s13293-026-00825-9)
Supplement: Supplementary file 1 — Supplementary Materials 1 [file 13293_2026_825_MOESM1_ESM.docx]

**Table S1:** Demographic and clinical data of Long COVID patients and controls disaggregated by sex.

|  | **Controls** | | **Long COVID** | |
| --- | --- | --- | --- | --- |
|  | **Female**  **(n = 19)** | **Male**  **(n = 7)** | **Female**  **(n= 24)** | **Male**  **(n= 10)** |
| **Age categories n (%)** |  |  |  |  |
| <30 | 5 (26.3%) | 0 | 0 | 0 |
| 30-50 | 5 (26.3%) | 1 (14.3%) | 10 (41.7%) | 2 (20%) |
| 51-60 | 4 (21%) | 4 (57.1%) | 9 (37.5%) | 6 (60%) |
| 61-70 | 4 (21%) | 2 (28.6%) | 3 (12.5%) | 0 |
| >70 | 1 (5.3%) | 0 | 2 (8.3%) | 2 (20%) |
| **Ethnicity n (%)** |  |  |  |  |
| Black | 0 | 0 | 4 (16.7%) | 3 (30%) |
| White | 19 (100%) | 7 (100%) | 20 (83.3%) | 7 (70%) |
| **Comorbidities n (%)** |  |  |  |  |
| Digestive System | 1 (5.3%) | 0 | 2 (8.3%) | 0 |
| Neurological System | 1 (5.3%) | 0 | 2 (8.3%) | 0 |
| Circulatory System | 6 (31.6%) | 3 (42.9%) | 7 (29.2%) | 2 (20%) |
| Respiratory System | 0 | 2 (28.6%) | 4 (16.7%) | 2 (20%) |
| Musculoskeletal System | 1 (5.3%) | 0 | 1 (4.2%) | 0 |
| Urinary tract | 0 | 0 | 1 (4.2%) | 0 |
| Genital tract | 0 | 2 (28.6%) | 1 (4.2%) | 0 |
| Endocrine, Metabolic and Nutritional | 8 (42.1%) | 6 (85.7%) | 18 (75%) | 7 (70%) |
| Psychological | 3 (15.8%) | 4 (57.1%) | 11 (45.8%) | 5 (50%) |
| Blood, Hematopoietic, Lymphatic and Spleen | 0 | 0 | 1 (4.2%) | 0 |
| None | 8 (42.1%) | 1 (14.3%) | 3 (12.5%) | 1 (10%) |
| **Vaccination Status and Doses n (%)** |  |  |  |  |
| Doses |  |  |  |  |
| 0 | 1 (5.3%) | 1 (14.3%) | 1 (4.2%) | 1 (10%) |
| 1 | 2 (10.5%) | 0 | 2 (8.3%) | 0 |
| 2 | 0 | 0 | 7 (29.2%) | 3 (30%) |
| 3 | 6 (31.6%) | 2 (28.6%) | 7 (29.2%) | 2 (20%) |
| 4 | 8 (42.1%) | 3 (42.9%) | 4 (16.6%) | 3 (30%) |
| 5 | 2 (10.5%) | 1 (14.3%) | 1 (4.2%) | 1 (10%) |
| 6 | 0 | 0 | 2 (8.3%) | 0 |
| Vaccine |  |  |  |  |
| Corminaty | 18 (94.7%) | 5 (71.4%) | 14 (58.3%) | 3 (30%) |
| Janssen | 0 | 0 | 3 (12.5%) | 3 (30%) |
| Spikevax | 0 | 0 | 4 (16.6%) | 1 (10%) |
| Vaxzevria | 0 | 1 (14.3%) | 4 (16.6%) | 2 (20%) |
| **Oxygen Support n (%)** |  |  |  |  |
| Yes | - | - | 1 (4.2%) | 0 |
| No | - | - | 23 (95.8%) | 0 |
| **Pneumonia n (%)** |  |  |  |  |
| Yes | - | - | 1 (4.2%) | 3 (30%) |
| No | - | - | 23 (95.8%) | 7 (70%) |
| **COVID-19 hospitalization n (%)** |  |  |  |  |
| Yes | - | - | 1 (4.2%) | 1 (10%) |
| No | - | - | 23 (95.8%) | 9 (90%) |

**Table S2:** Comorbidities divided by health system categories for controls and LC patients disaggregated by sex.

| **Health System Category** | **Controls** | | **Long COVID** | |
| --- | --- | --- | --- | --- |
|  | **Female**  **(n = 19)** | **Male**  **(n = 7)** | **Female**  **(n = 24)** | **Male**  **(n = 10)** |
| **Digestive System n (%)** |  |  |  |  |
| Ulcerative colitis | 0 | 1 (14%) | 0 | 0 |
| Functional alteration of the stomach | 0 | 0 | 1 (4%) | 0 |
| Heartburn | 0 | 0 | 1 (4%) | 0 |
| Malignant neoplasm of the colon/rectum | 0 | 0 | 1 (4%) | 0 |
| **Neurological System n (%)** |  |  |  |  |
| Congenital neurological malformation | 1 (5%) | 0 | 0 | 0 |
| Migraine | 0 | 0 | 2 (8%) | 0 |
| **Circulatory System n (%)** |  |  |  |  |
| Hypertension | 6 (32%) | 3 (43%) | 7 (29%) | 2 (20%) |
| Cerebrovascular disease | 0 | 1 (14%) | 0 | 0 |
| Ischemia heart disease with angina | 0 | 0 | 1 (4%) | 0 |
| **Respiratory System n (%)** |  |  |  |  |
| Asthma | 0 | 1 (14%) | 2 (8%) | 0 |
| Sinusitis | 0 | 1 (14%) | 1 (4%) | 1 (10%) |
| Chronic obstructive pulmonary disease | 0 | 1 (14%) | 1 (4%) | 0 |
| Respiratory Disease | 0 | 0 | 0 | 1 (10%) |
| **Musculoskeletal System n (%)** |  |  |  |  |
| Musculoskeletal system disease | 0 | 1 (14%) | 0 | 0 |
| Rheumatoid Arthritis | 0 | 0 | 1 (4%) | 0 |
| Osteoporosis | 0 | 0 | 1 (4%) | 0 |
| **Urinary tract n (%)** |  |  |  |  |
| Urinary stone | 0 | 0 | 1 (4%) | 0 |
| **Genital tract n (%)** |  |  |  |  |
| Malignant neoplasm of the prostate | 0 | 1 (14%) | 0 | 0 |
| Benign prostatic hypertrophy | 0 | 1 (14%) | 0 | 0 |
| Excessive menstruation | 0 | 0 | 1 (4%) | 0 |
| **Endocrine, Metabolic and Nutritional n (%)** |  |  |  |  |
| Alteration of lipid metabolism | 1 (5%) | 4 (57%) | 10 (42%) | 4 (40%) |
| Overweight | 3 (16%) | 3 (43%) | 5 (21%) | 1 (10%) |
| Obesity | 4 (21%) | 2 (29%) | 8 (33%) | 6 (60%) |
| Type 2 Diabetes | 1 (5%) | 1 (14%) | 2 (8%) | 3 (30%) |
| Hypothyroidism | 0 | 1 (14%) | 3 (13%) | 0 |
| Gout | 0 | 0 | 0 | 1 (10%) |
| **Psychological n (%)** |  |  |  |  |
| Depression | 0 | 2 (29%) | 7 (29%) | 1 (10%) |
| Sleep disturbance | 0 | 1 (14%) | 2 (8%) | 2 (20%) |
| Tobacco abuse | 2 (11%) | 1 (14%) | 4 (17%) | 1 (10%) |
| Affective psychosis | 1 (5%) | 0 | 1 (4%) | 0 |
| Anxiety | 0 | 0 | 1 (4%) | 1 (10%) |
| **Blood, Hematopoietic, Lymphatic and Spleen n (%)** |  |  |  |  |
| Lymphadenitis | 0 | 0 | 1 (4%) | 0 |

**Table S3:** Symptoms of LC patients divided by health system categories disaggregated by sex.

| **Health System category** | **Female**  **(n = 24)** | **Male**  **(n = 10)** |
| --- | --- | --- |
| **Neurocognitive n (%)** |  |  |
| Concentration problems | 19 (79%) | 5 (50%) |
| Anxiety | 17 (71%) | 4 (40%) |
| Oblivion | 17 (71%) | 6 (60%) |
| Insomnia | 16 (67%) | 8 (80%) |
| Loss of interest | 16 (67%) | 1 (10%) |
| Changing behaviour | 15 (63%) | 1 (10%) |
| Decreased/slowed movement | 13 (54%) | 0 |
| Depressive mood | 13 (54%) | 1 (10%) |
| Fainting | 2 (8%) | 0 |
| Hallucinations | 2 (8%) | 0 |
| **Neurosensory n (%)** |  |  |
| Dizziness | 15 (63%) | 7 (70%) |
| Balance changes | 14 (58%) | 2 (20%) |
| Numbness | 12 (50%) | 4 (40%) |
| Persistent headache | 12 (50%) | 3 (30%) |
| Decrease/absence/change in taste | 12 (50%) | 1 (10%) |
| Decrease/absence/change in smell | 10 (48%) | 1 (10%) |
| Vision changes | 8 (33%) | 4 (40%) |
| Tinnitus | 6 (25%) | 1 (10%) |
| Limb spasms | 7 (29%) | 1 (10%) |
| Hearing changes | 5 (21%) | 0 |
| Tremor | 3 (13%) | 1 (10%) |
| **Respiratory System/ Cardiothoracic n (%)** |  |  |
| Chest pain | 10 (42%) | 3 (30%) |
| Dyspnea | 10 (42%) | 3 (30%) |
| Pain when breathing | 9 (38%) | 1 (10%) |
| Persistent dry cough | 4 (17%) | 4 (40%) |
| **Digestive System n (%)** |  |  |
| Stomach pain | 9 (38%) | 2 (20%) |
| Nausea/vomit | 5 (21%) | 2 (20%) |
| Constipation | 6 (25%) | 4 (40%) |
| Change in swallowing | 4 (17%) | 1 (10%) |
| Diarrhea | 3 (13%) | 2 (20%) |
| **Musculoskeletal System/ Fatigue n (%)** |  |  |
| Persistent fatigue | 21 (88%) | 5 (50%) |
| Drowsiness | 18 (75%) | 6 (60%) |
| Persistent muscle pain | 13 (54%) | 5 (50%) |
| Weakness in legs/arms | 11 (46%) | 4 (40%) |
| Malaise after exercise | 9 (38%) | 3 (30%) |
| Muscle stiffness | 6 (25%) | 1 (10%) |
| Inability to move | 3 (13%) | 0 |
| **Dermatological/ Sexual/ Others n (%)** |  |  |
| Palpitation | 9 (38%) | 2 (20%) |
| Weight loss | 6 (25%) | 0 |
| Sexual dysfunction | 5 (21%) | 2 (20%) |
| Changing gait/falls | 4 (17%) | 1 (10%) |
| Fever | 4 (17%) | 1 (10%) |
| Toe injuries | 3 (13%) | 0 |
| Skin rash | 2 (8%) | 0 |
| Dysmenorrhea | 2 (8%) | 0 |
| Difficulty urinating | 1 (4%) | 1 (10%) |
| Peripheral edema | 1 (4%) | 1 (10%) |

**Table S4:** Comorbidity and age association with LC status by Fisher’s Exact Test and by Logistic Regression, respectively.

|  | **p value** |
| --- | --- |
| Endocrine, Metabolic and Nutritional | 0.172 |
| Circulatory System | 0.575 |
| Psychological | 0.18 |
| Respiratory System | 0.446 |
| Age* | 0.097 |

*Logistic regression

**Table S5:** Logistic regression for symptomatology unadjusted and adjusted to sex, age and time since infection.

|  | **p value^+^** | **Sex (p value)** | **Age (p value)** | **Time since infection (p value)** |
| --- | --- | --- | --- | --- |
| Persistent fatigue | 0.0002 | 0.0204 | 0.030 | 0.239 |
| Concentration problems | 0.313 | 0.109 | 0.622 | 0.561 |
| Insomnia | 0.389 | 0.571 | 0.842 | 0.144 |
| Drowsiness | 0.319 | 0.348 | 0.222 | 0.466 |
| Oblivion | 0.685 | 0.480 | 0.358 | 0.817 |
| Dizziness | 0.926 | 0.609 | 0.796 | 0.606 |
| Anxiety | 0.065 | 0.136 | 0.559 | 0.088 |
| Persistent muscle pain | 0.533 | 0.658 | 0.171 | 0.906 |
| Loss of interest | 0.009 | 0.12 | 0.499 | 0.397 |
| Changing behaviour | 0.021 | 0.014 | 0.337 | 0.909 |
| Numbness | 0.354 | 0.380 | 0.375 | 0.121 |
| Balance changes | 0.124 | 0.124 | 0.571 | 0.162 |
| Weakness in legs/arms | 0.569 | 0.605 | 0.973 | 0.189 |
| Persistent headache | 0.530 | 0.215 | 0.435 | 0.433 |
| Depressive mood | 0.021 | 0.027 | 0.129 | 0.658 |
| Decreased/slowed movement | 0.005 | 0.999 | 0.364 | 0,757 |
| Decrease/absence/change in taste | 0.110 | 0.061 | 0.991 | 0.462 |
| Dyspnea | 0.298 | 0.319 | 0.180 | 0.167 |
| Chest pain | 0.682 | 0.421 | 0.786 | 0.308 |
| Malaise after exercise | 0.817 | 0.595 | 1 | 0.401 |
| Vision changes | 0.688 | 0.528 | 0.367 | 0.386 |
| Decrease/absence/change in smell | 0.125 | 0.109 | 0.641 | 0.230 |
| Stomach pain | 0.165 | 0.554 | 0.078 | 0.537 |
| Palpitation | 0.643 | 0.288 | 0.436 | 0.942 |
| Pain when breathing | 0.136 | 0.097 | 0.790 | 0.149 |
| Constipation | 0.488 | 0.432 | 0.269 | 0.704 |

^+^Bonferroni adjusted p < 0.002

**Table S6:** Linear regression for CD8 T cell populations unadjusted and adjusted to sex, age and time since infection.

|  | **p value^+^** | **Sex (p value)** | **Age (p value)** | **Time since infection (p value)** | **LC status (p value)** |
| --- | --- | --- | --- | --- | --- |
| % CD8 T cells | 0.038 | 0.771 | 0.031 | 0.788 | 0.018 |
| % CD8^+^ Granzyme B^+^ cells | 0.019 | 0.353 | 0.008 | 0.372 | 0.184 |
| % CD8^+^ Granzyme K^+^ cells | 0.35 | 0.411 | 0.114 | 0.911 | 0.014 |
| % CD8^+^ Perforin^+^ cells | 0.088 | 0.250 | 0.379 | 0.04 | 0.48 |
| % CD8^+^ NKG2D^+^ cells | 0.628 | 0.473 | 0.901 | 0.919 | 0.247 |

^+^Bonferroni adjusted p < 0.01

**Table S7:** Linear regression for NK cell populations unadjusted and adjusted to sex, age and time since infection.

|  | **p value^+^** | **Sex (p value)** | **Age (p value)** | **Time since infection (p value)** | **LC status (p value)** |
| --- | --- | --- | --- | --- | --- |
| % CD56 T cells | 0.234 | 0.374 | 0.346 | 0.263 | 0.280 |
| % CD56^+^ Granzyme B^+^ cells | 0.479 | 0.377 | 0.200 | 0.459 | 0.931 |
| % CD56^+^ Granzyme K^+^ cells | 0.318 | 0.472 | 0.306 | 0.541 | 0.258 |
| % CD56^+^ Perforin^+^ cells | 0.319 | 0.657 | 0.594 | 0.056 | 0.561 |
| % CD56^+^ NKG2D^+^ cells | 0.451 | 0.467 | 0.806 | 0.126 | 0.472 |

^+^Bonferroni adjusted p < 0.01

**Table S8:** Linear regression for inflammatory plasma markers unadjusted and adjusted to sex, age and time since infection.

|  | **p value^+^** | **Sex (p value)** | **Age (p value)** | **Time since infection (p value)** | **LC status (p value)** |
| --- | --- | --- | --- | --- | --- |
| TNF-α (pg/ml) | 0.532 | 0.527 | 0.273 | 0.955 | 0.291 |
| IL-10 (pg/ml) | 0.110 | 0.219 | 0.763 | 0.046 | 0.081 |
| sCD40L (pg/ml) | 0.157 | 0.344 | 0.780 | 0.239 | 0.266 |
| sFAS (pg/ml) | 0.215 | 0.348 | 0.116 | 0.317 | 0.595 |
| Perforin (pg/ml) | 0.148 | 0.364 | 0.317 | 0.068 | 0.015 |
| Granzyme A (pg/ml) | 0.013 | 0.097 | 0.002 | 0.636 | 0.848 |
| Granzyme B (pg/ml) | 0.737 | 0.310 | 0.697 | 0.300 | 0.661 |

^+^Bonferroni adjusted p < 0.007

**Table S9:** Effect sizes determined by correlation coefficient r for Mann-Whitney tests.

| **Figure** | **Effect size (r)** | **Classification** |
| --- | --- | --- |
| 1C | 0.207 | Small |
| 1D Ctr | 0.187 | Small |
| 1E | 0.305 | Medium |
| 1G | 0.195 | Small |
| 1H | 0.099 | Small |
| 2A | 0.259 | Small |
| 2B Female | 0.415 | Medium |
| 4E Female | 0.480 | Medium |
| 4F Female | 0.120 | Small |
| 4I Female | 0.200 | Small |
| 4I Male | 0.276 | Small |
| 4J Male | 0.242 | Small |
| 5A Female | 0.357 | Medium |
| 5A Male | 0.761 | Large |
| 5B Female | 0.078 | Small |
| 5B Male | 0.365 | Medium |
| 5C Female | 0.408 | Medium |
| 5D Female | 0.342 | Medium |
| 5D Male | 0.275 | Small |
| 5E Female | 0.365 | Medium |
| 5F Female | 0.000 | Small |
| 5F Male | 0.061 | Small |
| 5G Female | 0.271 | Small |
| S3B | 0.173 | Small |
| S3E | 0.162 | Small |
| S3F | 0.028 | Small |
| S3G | 0.259 | Small |
| S3H | 0.261 | Small |
| S3I | 0.095 | Small |
| S3J | 0.130 | Small |
| S3K | 0.032 | Small |
| S3L | 0.184 | Small |
| S3M | 0.164 | Small |
| S3N | 0.142 | Small |
| S4C | 0.271 | Small |
| S4H | 0.403 | Medium |
| S4I | 0.053 | Small |
| S4L | 0.361 | Medium |
| S5A | 0.466 | Medium |
| S5B | 0.060 | Small |
| S5C | 0.424 | Medium |
| S5D | 0.323 | Medium |
| S5E | 0.400 | Medium |
| S5F | 0.018 | Small |
| S5G | 0.133 | Small |
| S5H | 0.230 | Small |
| S5I | 0.013 | Small |
| S5J | 0.085 | Small |
| S5K | 0.160 | Small |
| S5L | 0.095 | Small |
| S5M | 0.041 | Small |

**Table S10:** Effect sizes determined by Cohen’s d for unpaired t tests.

| **Figure** | **Effect size (d)** | **Classification** |
| --- | --- | --- |
| 1D LC | 0.338 | Medium |
| 1F | 0.747 | Medium |
| 2B Male | 0.459 | Medium |
| 3A | 0.904 | Medium |
| 4A Female | 0.367 | Medium |
| 4A Male | 1.545 | Large |
| 4B Female | 0.402 | Medium |
| 4B Male | 0.331 | Medium |
| 4C Female | 0.738 | Medium |
| 4C Male | 0.978 | Large |
| 4D Female | 0.051 | Small |
| 4D Male | 0.298 | Small |
| 4E Male | 0.407 | Medium |
| 4F Male | 0.700 | Medium |
| 4G Female | 0.039 | Small |
| 4G Male | 0.182 | Small |
| 4H Female | 0.272 | Small |
| 4H Male | 1.014 | Large |
| 4J Female | 0.002 | Small |
| 5C Male | 0.959 | Large |
| 5E Male | 1.002 | Large |
| 5G Male | 0.777 | Medium |
| S3A | 0.453 | Medium |
| S3C | 0.402 | Medium |
| S3D | 0.042 | Small |
| S4A | 0.631 | Medium |
| S4B | 0.147 | Small |
| S4D | 0.194 | Small |
| S4E | 0.390 | Medium |
| S4F | 0.831 | Large |
| S4G | 0.054 | Small |
| S4J | 0.084 | Small |
| S4K | 0.485 | Medium |
| S4M | 0.007 | Small |
