# Supplementary figures and images for: Long-term sex differences in symptoms and immune profile in long COVID
Source: Biol Sex Differ. 2026 Jan 27;17:32. doi: 10.1186/s13293-026-00825-9 (PMC12918094; doi:10.1186/s13293-026-00825-9)

Figure Supplemental 1

A

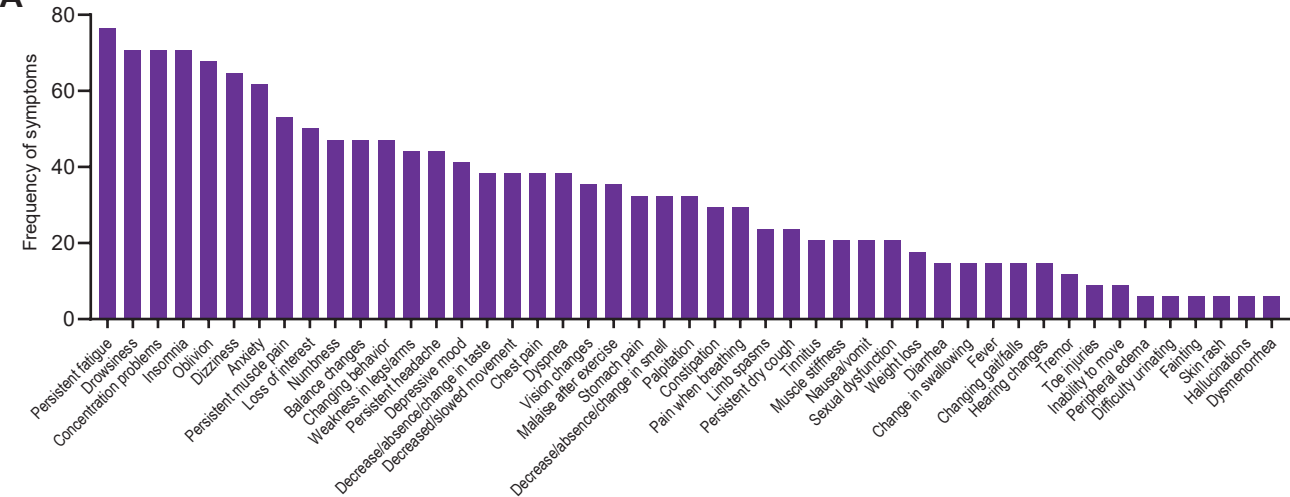

B

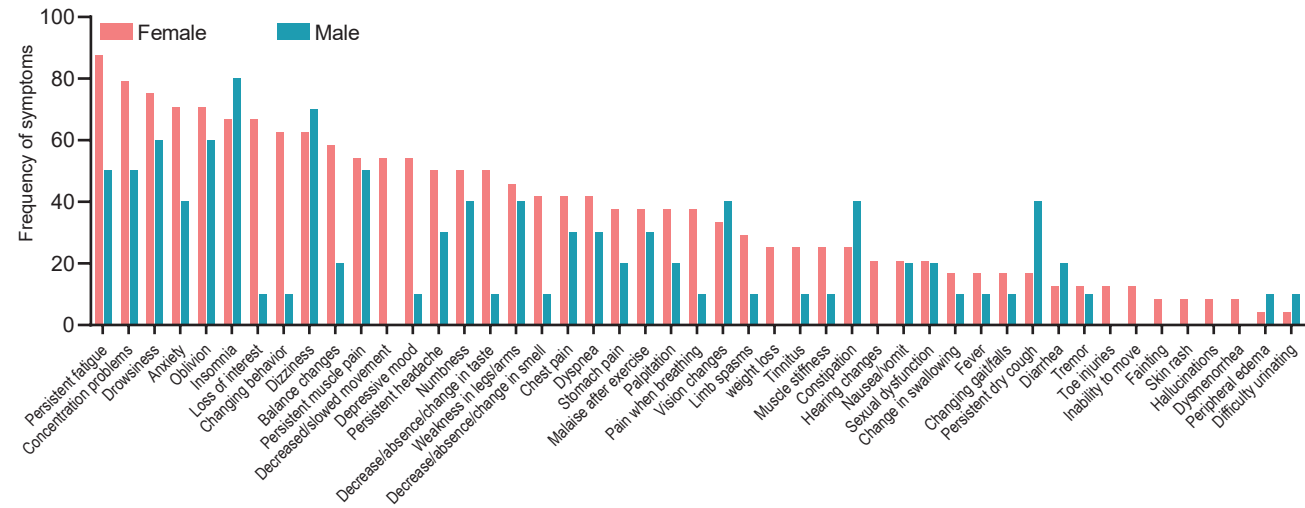

C

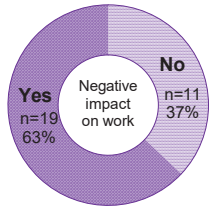

Supplement: Supplementary file 2 — Supplementary Materials 2: Fig. S1 Frequency of symptoms in Long COVID patients. (A) Frequency of symptoms of LC patients. (B) Frequency of symptoms disaggregated by sex (females, pink; males, blue). (C) Work-related impacts of LC [file 13293_2026_825_MOESM2_ESM.pdf]

Figure Supplemental 2

A

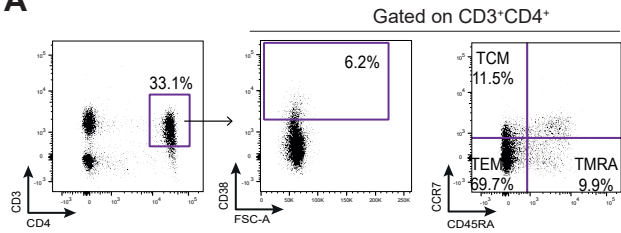

B

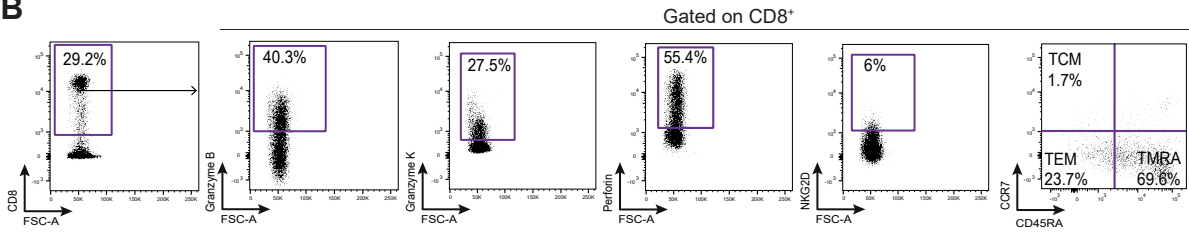

C

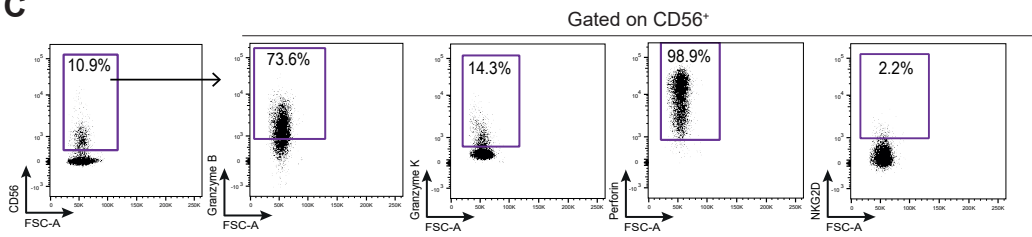

D

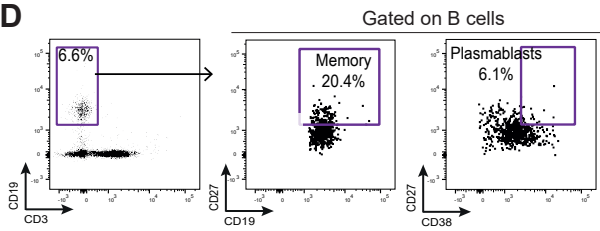

Supplement: Supplementary file 3 — Supplementary Materials 3: Fig. S2 Flow cytometric gating strategy. (A) Gating strategy for activated and memory CD4+ T cell subsets. (B) Gating strategy for Granzyme B+, Granzyme K+, Perforin+ and NKG2D+ CD8+ T cells and memory CD8+ T cell subsets. (C) Gating strategy for Granzyme B+, Granzyme K+, Perforin+ and NKG2D+ CD56+ cells. (D) Gating strategy for CD19+CD3−CD27+ and CD19+CD3−CD27+CD38+ B cells [file 13293_2026_825_MOESM3_ESM.pdf]

Figure Supplemental 3

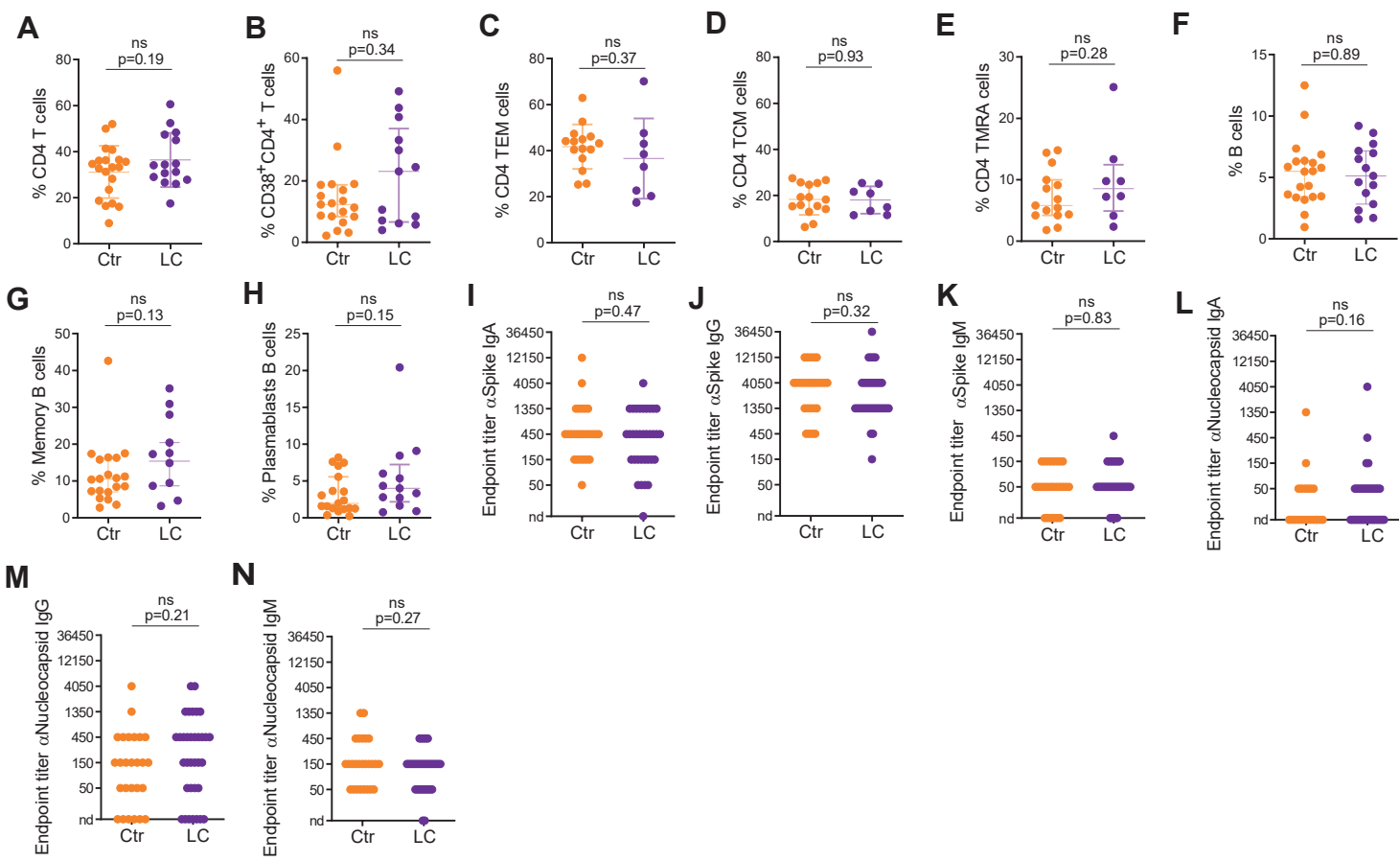

Supplement: Supplementary file 4 — Supplementary Materials 4: Fig. S3 Humoral and cellular responses in Long COVID. (A) Frequency of CD4+ T cells in Ctr (n = 20) and LCs (n = 15). (B) Frequency of CD4+ CD38+ T cells in Ctr (n = 19) and LCs (n = 13). (C) Frequency of CD4+ T effector memory (TEM) cells in Ctr (n = 15) and LCs (n = 8). (D) Frequency of CD4+ T central memory (TCM) cells in Ctr (n = 15) and LCs (n = 8). (E) Frequency of CD4+ TMRA cells in Ctr (n = 15) and LCs (n = 8). (F) Frequency of circulating B cells in Ctr (n = 20) and LCs (n = 15). (G) Frequency of memory B cells in Ctr (n = 20) and LCs (n = 11). (H) Frequency of plasmablasts B cells in Ctr (n = 19) and LCs (n = 13) (I) Anti-spike IgA endpoint titer (Ctr n = 26; n = 34). (J) Anti-spike IgG endpoint titer (Ctr n = 26; LC n = 34). (K) Anti-spike IgM endpoint titer (Ctr n = 26; LC n = 34). (L) Anti-Nucleocapsid IgA endpoint titer (Ctr n = 26; LC n = 34). (M) Anti-spike IgG endpoint titer (Ctr n = 26; LC n = 34). (N) Anti-spike IgM endpoint titer (Ctr n = 26; LC n = 34). Data represents mean ± SD for parametric tests, or median ± IQR for nonparametric tests. nd: not detectable; p values ns, not significant determined by parametric unpaired t test (A, C, D) and by non-parametric Mann-Whitney test (B, E-N). Effect sizes for all graphs are reported in Tables S9 and S10. [file 13293_2026_825_MOESM4_ESM.pdf]

**Figure Supplemental 4**

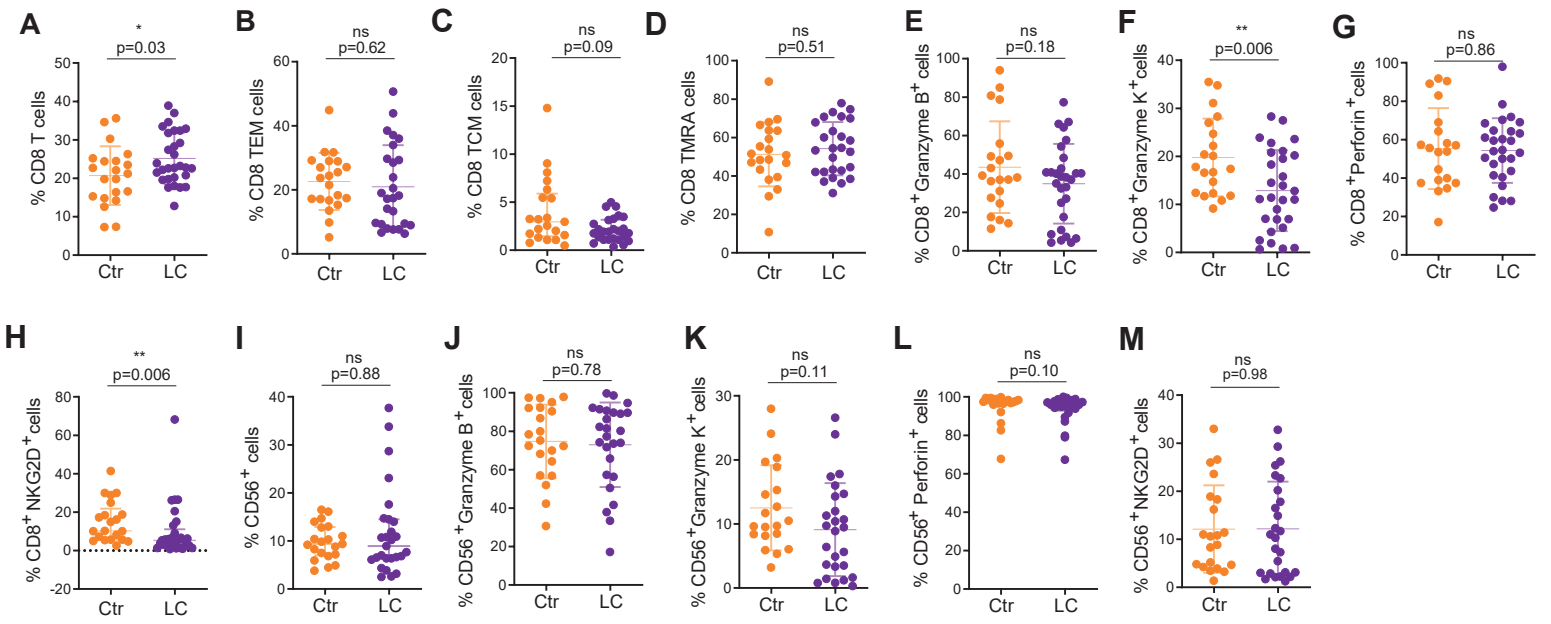

Supplement: Supplementary file 5 — Supplementary Materials 5: Fig. S4 CD8⁺ T cell and NK cell characterization in controls and Long COVID patients. (A) Frequency of CD8+ T cells in Ctr (n = 21) and LC (n = 29). (B) Frequency of CD8+ T effector memory (TEM) cells in Ctr (n = 21) and LCs (n = 26). (C) Frequency of CD8+ T central memory (TCM) cells in Ctr (n = 21) and LCs (n = 26). (D) Frequency of CD8+ TMRA cells in Ctr (n = 21) and LCs (n = 26). (E) Frequency of CD8+Granzyme B+ cells in Ctr (n = 21) and LC (n = 29). (F) Frequency of CD8+Granzyme K+ cells in Ctr (n = 20) and LC (n = 29). (G) Frequency of CD8Perforin+ cells in Ctr (n = 20) and LC (n = 28). (H) Frequency of CD8+ NKG2D+ cells in Ctr (n = 21) and LC (n = 29). (I) Frequency of CD56+ cells in Ctr (n = 21) and LC (n = 27). (J) Frequency of CD56+Granzyme B+ cells in Ctr (n = 21) and LC (n = 26). (K) Frequency of CD56+Granzyme K+ cells in Ctr (n = 20) and LC (n = 26). (L) Frequency of CD56+Perforin+ cells in Ctr (n = 20) and LC (n = 25). (M) Frequency of CD56+ NKG2D+ cells in Ctr (n = 21) and LC (n = 26). Data represents mean ± SD for parametric tests, or median ± IQR for nonparametric tests. p values *p < 0.05, **p < 0.01; ns, not significant determined by parametric unpaired t test (A, B, D-G, J, K, M) and by non-parametric Mann–Whitney test (C, H, I, L). Effect sizes for all graphs are reported in Tables S9 and S10 [file 13293_2026_825_MOESM5_ESM.pdf]

**Figure Supplemental 5**

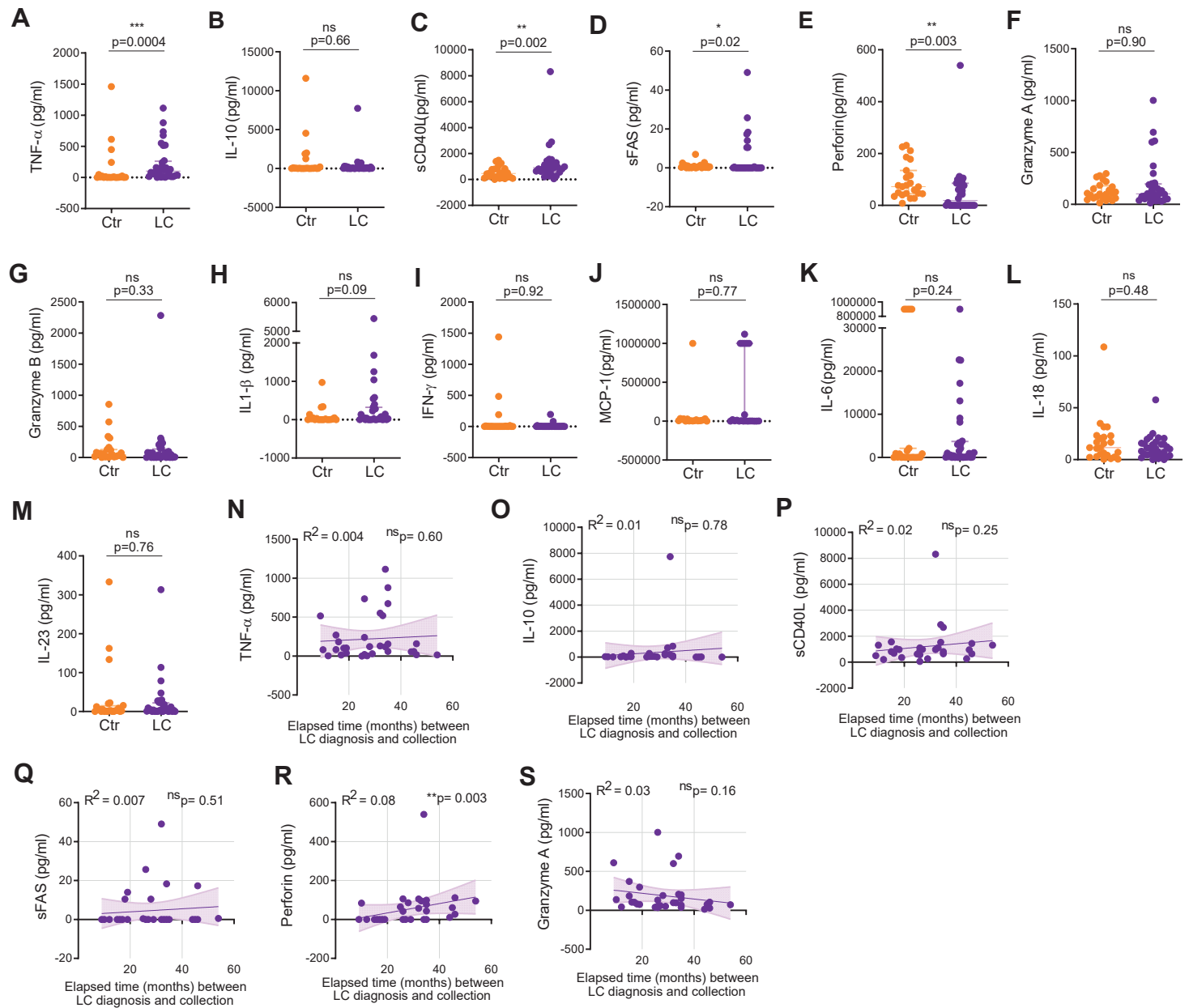

Supplement: Supplementary file 6 — Supplementary Materials 6: Fig. S5 Inflammatory plasma profile in controls and persistent Long COVID patients (A) Plasma concentration of TNF-α (pg/ml) in Ctr (n = 22) and LC (n = 32). (B) Plasma concentration of IL-10 (pg/ml) in Ctr (n = 24) and LC (n = 32). (C) Plasma concentration of sCD40L (pg/ml) in Ctr (n = 24) and LC (n = 30). (D) Plasma concentration of sFAS (pg/ml) in Ctr (n = 24) and LC (n = 32). (E) Plasma concentration of Perforin (pg/ml) in Ctr (n = 24) and LC (n = 32). (F) Plasma concentration of Granzyme A (pg/ml) in Ctr (n = 24) and LC (n = 32). (G) Plasma concentration of Granzyme B (pg/ml) in Ctr (n = 24) and LC (n = 32). (H) Plasma concentration of IL-1β (pg/ml) in Ctr (n = 20) and LC (n = 32). (I) Plasma concentration of IFN-$\upgamma$ (pg/ml) in Ctr (n = 24) and LC (n = 32). (J) Plasma concentration of MCP-1 (pg/ml) in Ctr (n = 18) and LC (n = 21). (K) Plasma concentration of IL-6 (pg/ml) in Ctr (n = 23) and LC (n = 32). (L) Plasma concentration of IL-18 (pg/ml) in Ctr (n = 24) and LC (n = 32). (M) Plasma concentration of IL-23 (pg/ml) in Ctr (n = 24) and LC (n = 32). (N) Correlation between plasma TNF-α concentration (pg/mL) and elapsed time (months) between LC diagnosis and sample collection (n = 32). (O) As in H for IL-10 (n = 32). (P) As in H for sCD40L (n = 30). (Q) As in H for sFAS (n = 32). (R) As in H for Perforin (n = 32). (S) As in H for Granzyme A (n = 32). Data represents median ± IQR for nonparametric tests. p values *p < 0.05, **p < 0.01, ***p < 0.0001; ns, not significant determined by non-parametric Mann-Whitney test (A-M). Spearman correlation (N-S). Effect sizes for all graphs are reported in Table S9. [file 13293_2026_825_MOESM6_ESM.pdf]
